# Inhibition of translation and immune responses by the virulence factor Nsp1 of SARS-CoV-2

**DOI:** 10.1038/s41392-020-00350-0

**Published:** 2020-10-09

**Authors:** Kendra R. Vann, Adam H. Tencer, Tatiana G. Kutateladze

**Affiliations:** Department of Pharmacology, University of Colorado School of Medicine, Aurora, CO 80045 USA

**Keywords:** Structural biology, Pathogenesis

Structural analysis of SARS-CoV-2 virulence factors is crucial for the development of vaccines and anti-viral therapeutics. Recent cryo-EM structures of the viral protein Nsp1 bound to the human ribosomal machinery shed light onto the mechanism of translational shutdown and blockage of host immune response.^1^

The global pandemic of COVID-19 has been ravaging the world for several months and continues posing an enormous threat to public health. This outbreak is the most serious pandemic occurred in the last 2 decades with morbidity reaching 18 million as of August 2020. The causative agent of COVID-19—Severe Acute Respiratory Syndrome CoronaVirus-2 (SARS-CoV-2)—belongs to the group of highly pathogenic beta-coronaviruses that also includes SARS-CoV and MERS-CoV, which caused respiratory diseases in 2002 and 2012.

**Fig. 1:**
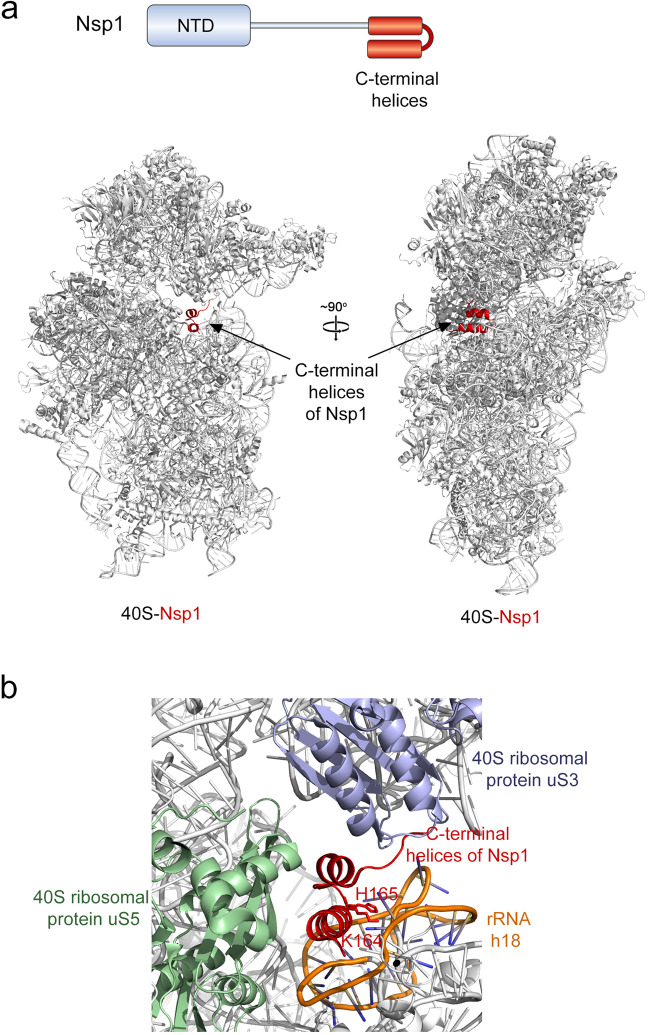
Structural basis for binding of the SARS-CoV-2 Nsp1 protein to the ribosomal 40S subunit. **a** Domain architecture of the SARS-CoV-2 Nsp1 protein (top). The N-terminal domain (NTD) and the C-terminal α-helices are labeled and colored light blue and red, respectively. Cryo-EM structure of the Nsp1 protein bound to 40S (PDB 6zlw) (bottom). The two C-terminal α-helices of Nsp1 (red) insert into the mRNA entry channel of 40S (gray). The resolution of the NTD region of Nsp1 in the cryo-EM density map was insufficient to unambiguously identify this domain. **b** Close view of the interface of the 40S:Nsp1 complex (PDB 6zlw). The two C-terminal α-helices of Nsp1 (red) make contacts with ribosomal rRNA h18 (orange phosphate backbone and blue nucleotide bases) and with the ribosomal proteins uS3 (light blue) and uS5 (light green). The K164 and H165 residues of the KH motif of Nsp1 are shown as red sticks

The SARS-CoV-2 and SARS-CoV viruses are closely related and share ~80% nucleotide identity in their single-stranded RNA genomes. The SARS-CoV-2 genome encodes four structural proteins, such as Spike (S), Envelope (E), Membrane (M) and Nucleocapsid (N), several non-structural proteins (Nsp), and the open-reading frame (ORF1a and ORF1ab) precursor polyproteins that are proteolytically cleaved in the host cells producing an additional set of Nsps, including Nsp1 (Fig. 1a). Nsp1 of SARS-CoV has been shown to act as a major virulence factor. It binds to the small ribosomal subunit, inhibiting production of host proteins by blocking messenger RNA (mRNA) translation and promoting degradation of host mRNA. Importantly, by inducing a near complete shutdown of translation of host proteins, including interferons, Nsp1 destroys the defense mechanisms of the innate immune system and facilitates viral replication. Owning to its crucial role in immune response dysregulation, Nsp1 of SARS-CoV-2 may represent an attractive pharmacological target in the fight against COVID-19.

Recent structural studies of Nsp1 of SARS-CoV-2 by Thoms et al.^1^ and Schubert et al.^2^ illuminated the molecular mechanism by which Nsp1 binds to the human ribosomal machinery and can be instrumental in guiding the development of inhibitors and vaccines. Thoms et al.^1^ report that Nsp1 strongly associates with purified human ribosomal 40S subunit and 80S ribosomes, but not with actively translating polyribosomes and that the presence of Nsp1 precludes mRNA translation in vitro and in HEK293T cells. The authors also found that the C-terminal residues K164 and H165 of Nsp1, comprising the KH motif, which is conserved in SARS-CoV, are required for this interaction and inhibition of translation. Mutation of the KH motif reduces the ability of Nsp1 to associate with ribosomal subunits and block host mRNA translation.

To understand the molecular basis of the Nsp1 function, Thoms et al.^1^ determined the cryo-EM structures of Nsp1 in complex with human ribosomal 40S and 80S subunits. The structures were refined to a high 2.6 Å average resolution, allowing for detailed analysis of the complexes and elucidating key contacts and the overall mechanism underlying inhibition of mRNA translation by Nsp1. In the Nsp1:40S complex, two C-terminal α-helices of Nsp1 (colored red in Fig. 1a) insert into the mRNA entry channel. The first α-helix (residues 154–160 of Nsp1) interacts with the ribosomal proteins uS3 and uS5 (colored light blue and light green, respectively, in Fig. 1b), whereas the second α-helix (residues 166–179 of Nsp1) interacts with uS5 and ribosomal rRNA h18. The structure also reveals that the two helices are stabilized through hydrophobic interactions. The HK motif of Nsp1 is located in the loop between the two helices and forms important contacts with rRNA h18. Specifically, K164 of Nsp1 occupies the negatively charged pocket formed by the backbone phosphates of G625 and U630 of rRNA, whereas H165 is stacked between U607 and U630. Overall, a high degree of complementarity in electrostatics and shape between the two helices of Nsp1 and the mRNA channel of the ribosomal subunit allowed Nsp1 to act as a plug, which effectively obstructs the mRNA entry channel. Independently, based on the cryo-EM structure of Nsp1 bound to the ribosomal 40S subunit, Schubert et al.^2^ came to a similar conclusion that Nsp1 inhibits translation by sterically occluding the mRNA channel entrance and interfering with the binding of the host mRNA.

Blocking the host mRNA translation machinery impedes production of the proteins necessary for anti-viral defense and other normal cell functions. Thoms et al.^1^ show that Nsp1 almost completely prevents translation of interferons IFN-β and IFN-λ1 and interleukin-8, as well as interferon stimulated anti-viral factors. Mutation of the Nsp1 KH motif restored the innate immune response in infected cells, further pointing to the vital role of the Nsp1 C-terminal helices in disabling the host cell anti-viral defense system.

Considering the ability of Nsp1 to substantially downregulate the innate immune responses, this virulence factor could become another appealing drug target in addition to the Spike protein and the RNA polymerase Nsp12 of SARS-CoV-2.^3–5^ Identification of compounds that bind and impair the C-terminal helices of Nsp1 or the N-terminal domain of Nsp1 can be an important new treatment strategy. Further research is also needed to determine whether Nsp1 can be used for the development of a vaccine. Lastly, to better understand the SARS-CoV-2 pathogenesis, it is essential to identify the mechanism by which SARS-CoV-2 produces its own viral proteins, overcoming the Nsp1-mediated host mRNA translation shutdown.
